# The Role of Large Language Models in Hormonal Contraception Consultation

**DOI:** 10.3390/jcm15145543

**Published:** 2026-07-15

**Authors:** Iason Psilopatis, Julius Emons, Tibor A. Zwimpfer

**Affiliations:** 1 Department of Gynecology and Obstetrics, University Hospital Basel, 4031 Basel, Switzerland; 2 Department of Gynecology and Obstetrics, Universitätsklinikum Erlangen, Comprehensive Cancer Center Erlangen-EMN (CCC ER-EMN), Friedrich-Alexander-Universität Erlangen-Nürnberg (FAU), 91054 Erlangen, Germany; 3 Department of Biomedicine, University Hospital and University of Basel, 4031 Basel, Switzerland

**Keywords:** large language models, hormonal, contraception, consultation

## Abstract

**Background:** Large Language Models (LLMs) demonstrate promise in medical applications, but their performance in hormonal contraception consultation remains underexplored. **Objective:** To evaluate the accuracy and comprehensiveness of LLM-generated hormonal contraception counseling compared to evidence-based guidelines. **Methods:** Ten fictitious clinical case scenarios representing common contraceptive counseling situations were presented to Chat-GPT, Google Gemini, and OpenEvidence. Cases assessed medical eligibility screening, contraindication recognition, drug interactions, side effect management, and emergency contraception guidance. Two board-certified obstetrician–gynecologists independently evaluated the responses based on international clinical guidelines. **Results:** Across the ten predefined clinical scenarios, all three LLMs achieved high accuracy in identifying contraindications according to Medical Eligibility Criteria (MEC) and provided appropriate alternative contraceptive recommendations. Models demonstrated high proficiency in managing drug interactions, particularly the lamotrigine–estrogen interaction, and provided evidence-based side effect management strategies. However, the communication styles differed. Chat-GPT emphasized structured consultation and shared decision-making, Gemini provided practical action-oriented guidance, and OpenEvidence delivered concise evidence-focused summaries. **Conclusions:** In this limited set of standardized fictitious cases, the evaluated LLMs generally provided responses that were broadly aligned with selected guideline recommendations. Current LLMs require cautious deployment, given limitations in individualized assessment and health literacy optimization, and are best positioned as complementary educational tools rather than replacements for professional contraceptive counseling.

## 1. Introduction

Large Language Models (LLMs) have emerged as transformative tools in modern medicine, demonstrating remarkable capabilities across diverse clinical applications. These artificial intelligence systems, trained on vast amounts of medical literature and clinical data, can process natural language queries and generate contextually appropriate responses without task-specific training [1]. Current applications of LLMs in healthcare include clinical documentation through ambient audio analysis, automated summarization of patient records and imaging results, clinical decision support, patient communication via chatbots, administrative task automation, and research literature synthesis [2]. Recent studies have shown that advanced LLMs such as GPT-4 and Med-PaLM 2 can achieve accuracy rates exceeding 85% on medical licensing examination-style questions, with some models approaching or surpassing human physician performance in specific domains [3,4,5]. Despite these promising results, LLMs face important limitations including the generation of inaccurate but convincing information, biases in training data, privacy concerns, and the inability to perform physical examinations or accurately assess individual patient contexts [1,5].

In the field of gynecology and obstetrics, LLMs have demonstrated particular promise as tools for patient education and clinical support. These AI-driven systems provide continuous access to information on sensitive health topics such as menstruation, contraception, pregnancy, and childbirth, thereby strengthening patient knowledge and autonomy while reducing barriers related to stigma and embarrassment [6]. Studies evaluating LLMs in obstetrics and gynecology have shown that models like Chat-GPT or Google Gemini might provide accurate and complete responses to patient questions across various gynecologic topics, with some studies demonstrating that AI-generated responses are often comparable to those from human specialists in terms of accuracy and suitability for patient communication [7,8,9]. The integration of LLMs in gynecology offers opportunities to enhance the partnership-based dialog between patients and healthcare professionals by enabling patients to better prepare for medical consultations and ask informed questions [6,10]. However, these tools should serve as complementary resources rather than replacements for medical consultations, as they lack the ability to assess individual health conditions or support fully personalized medical decision-making [6].

Hormonal contraception consultation represents a critical component of reproductive healthcare that requires comprehensive patient counseling and individualized decision-making. Effective contraceptive counseling encompasses several essential elements such as reviewing the patient’s general thoughts, fears, and questions about birth control methods, or presenting all contraceptive options with information on efficacy, mechanism of action, safety, administration details, and side-effect profiles, under the consideration of medical eligibility criteria using standardized frameworks [11,12,13]. For combined hormonal contraceptives specifically, consultation must also address relevant contraindications including severe hypertension, thromboembolic disorders, certain cardiovascular conditions, migraines with aura, and specific liver diseases, though the prevalence of true medical contraindications among reproductive-aged women is relatively low at approximately 2–7% [11,14].

Although LLMs have demonstrated promising performance in a variety of medical applications and have increasingly been evaluated in obstetrics and gynecology, their role in hormonal contraception consultation remains insufficiently investigated. The present study aims to investigate the role of LLMs in providing hormonal contraception consultation, evaluating their capacity to deliver accurate, comprehensive, and patient-appropriate information comparable to that provided by clinical guidelines. Given the demonstrated potential of LLMs in medical question-answering and patient education, combined with the structured nature of contraceptive counseling guidelines, this research seeks to assess whether LLMs can serve as effective supplementary tools for contraceptive education and decision support.

## 2. Methods and Materials

### 2.1. Study Design

This study employed a cross-sectional comparative analysis to evaluate the performance of three leading, internationally used LLMs in providing hormonal contraception consultation. The queries were conducted in January 2026 using the standard, default public-use web browser interfaces of each platform via the investigators’ existing free-tier user accounts: Chat-GPT (OpenAI Free Tier), Google Gemini (Free Tier), and the production version of OpenEvidence available at that time. Each LLM was presented with identical fictitious clinical case scenarios in the English language and their responses were analyzed for accuracy, completeness, and adherence to evidence-based guidelines.

### 2.2. Case Development

Ten fictitious clinical case scenarios were developed to represent common and clinically significant situations encountered in hormonal contraception consultation. These cases were intentionally curated to provide a balanced and representative sample of contraceptive counseling situations, spanning both high-frequency clinical encounters and high-risk medical scenarios. Case development was informed by the American College of Obstetricians and Gynecologists (ACOG) Practice Bulletin No. 206 on the use of hormonal contraception in women with coexisting medical conditions and the Centers for Disease Control and Prevention (CDC) U.S. Selected Practice Recommendations for Contraceptive Use [15,16]. The cases were designed to assess LLM performance across multiple domains including medical eligibility screening, contraindication recognition, drug interaction counseling, side effect management, and emergency contraception guidance. All ten clinical vignettes were developed prospectively by the study investigators prior to initiating any testing or data collection within the LLM user interfaces. Before deploying the full protocol, a brief pilot test utilizing a separate, unlisted clinical scenario was conducted across the platforms. This pilot phase was designed solely to ensure that the open-ended phrasing (‘How would you consult this patient?’) successfully prompted comprehensive, structured counseling narratives rather than fragmented or binary responses. No iterative adjustments were made to the core ten clinical cases following this pilot phase.

The ten case scenarios included: (1) a 35-year-old active smoker with history of deep vein thrombosis requesting hormonal contraception; (2) a 29-year-old patient with systemic lupus erythematosus seeking contraceptive counseling; (3) a 23-year-old breastfeeding patient two weeks postpartum wishing to initiate hormonal contraception; (4) a 22-year-old patient with headaches accompanied by speech disturbances and motor weakness requesting contraception; (5) a 33-year-old patient with epilepsy on lamotrigine therapy seeking hormonal contraception; (6) a 33-year-old patient inquiring about backup contraception needs after levonorgestrel intrauterine device (LNG-IUD) insertion; (7) a 26-year-old woman presenting to the emergency department with ongoing spotting three weeks after copper intrauterine device (Cu-IUD) insertion; (8) a 22-year-old patient with persistent amenorrhea following etonogestrel implant insertion in August 2025; (9) a 34-year-old patient who missed two consecutive active pills containing 30 µg ethinyl estradiol; and (10) a 22-year-old patient who vomited one hour after taking ulipristal acetate emergency contraception (UPA-ECP) (Table 1).

These cases were specifically selected to evaluate LLM competency in recognizing absolute contraindications to combined hormonal contraceptives (Cases 1, 2, 4), understanding postpartum contraception timing and venous thromboembolism risk (Case 3), identifying clinically significant drug interactions (Case 5), providing accurate guidance on backup contraception requirements (Case 6), managing common side effects of long-acting reversible contraception (Cases 7, 8), counseling on missed pill protocols (Case 9), and advising on emergency contraception failure management (Case 10).

### 2.3. Data Collection

Each of the ten clinical case scenarios was presented to all three LLMs using standardized prompts. The prompts were formatted as direct clinical questions beginning with “A [age]-year-old patient…” followed by the relevant clinical information and concluding with “How would you consult this patient?” This open-ended format was chosen to allow LLMs to demonstrate their ability to provide comprehensive contraceptive counseling without leading questions or multiple-choice options. All queries were conducted during the same time period to minimize temporal variations in LLM performance due to model updates. Responses were recorded verbatim and stored for subsequent analysis. All ten clinical case scenarios were presented sequentially within a single, continuous chat session for each respective model. No system prompts, custom instructions, or underlying platform settings were modified prior to the session, and pre-existing browser history or active account memory features were left at their default states. No iterative or follow-up prompts were used to ensure standardization across all assessments.

### 2.4. Outcome Measures

The primary outcome measure was the accuracy and completeness of contraceptive counseling provided by each LLM compared to evidence-based guidelines. Responses were evaluated based on several key criteria: (1) correct identification of contraindications according to CDC Medical Eligibility Criteria (MEC) categories; (2) appropriate recommendation of alternative contraceptive methods when (combined) hormonal contraceptives were contraindicated; (3) accurate counseling regarding drug interactions and their clinical implications; (4) provision of evidence-based management strategies for common side effects and complications; (5) correct guidance on backup contraception requirements and missed pill protocols; and (6) appropriate emergency contraception counseling including management of treatment failure. Secondary outcome measures included assessment of the comprehensiveness of patient education provided, inclusion of relevant safety information and warning signs, discussion of shared decision-making principles, and overall quality of communication appropriate for patient counseling.

### 2.5. Data Analysis

Responses from each LLM were independently reviewed and scored by two board-certified obstetrician–gynecologists with over ten years of clinical experience in contraceptive counseling. Evaluators were blinded to the LLM source of each response. An evaluation rubric was developed based on current clinical practice guidelines from ACOG, CDC, the German Society of Gynecology and Obstetrics, and the Faculty of Sexual and Reproductive Healthcare [15,16,17,18]. For each of the ten clinical scenarios, predefined key counseling elements were established according to the guideline recommendations (Table 2). These included mandatory elements that were considered essential for guideline-concordant counseling, such as correct identification of contraindications, appropriate MEC classification, recommendation of safe contraceptive alternatives, accurate management advice, and recognition of clinically relevant drug interactions or emergency management protocols. Additional counseling elements, including patient education, discussion of patient preferences, practical counseling points, safety-net advice, and follow-up recommendations, were considered desirable but not essential for clinical correctness. Each response was compared directly with the corresponding guideline-based gold standard and classified as accurate when all mandatory elements were present, as complete when all mandatory and optional elements were correctly addressed, or as clinically unacceptable when one or more mandatory elements were missing or incorrect, unsafe recommendations were provided, or advice deviated from current evidence-based guidelines in a manner that could influence patient safety or clinical decision-making. The expert reviewers evaluated the responses as patient-directed counseling while assessing their clinical accuracy, completeness, and concordance with contemporary international guidelines. Descriptive analyses were performed by comparing each model against the predefined criteria across all case scenarios. Each response was independently assessed by both reviewers before consensus was reached. Whenever differences in interpretation occurred, the responses were jointly re-evaluated against the predefined guideline-based gold standard and discussed until agreement on the final classification was achieved. Because this was an exploratory feasibility study with only ten clinical scenarios, the evaluation was designed as a qualitative expert consensus process, and formal inter-rater agreement statistics were not prospectively planned.

## 3. Results

### 3.1. Clinical Accuracy and MEC Classification

Across all ten clinical cases, all three LLMs demonstrated high accuracy in identifying primary contraindications as defined by the USMEC and World Health Organization (WHO) MEC guidelines. For instance, in cases involving high-risk conditions like active smoking with a history of deep vein thrombosis (Case 1) or migraines with aura (Case 4), every model correctly identified combined hormonal contraceptives as USMEC/WHO MEC Category 4 (unacceptable health risk). While the models were universally correct in flagging these safety risks, OpenEvidence mirrored the gold standard’s concise technical terminology most closely. In contrast, Chat-GPT and Gemini frequently provided more expansive explanations, such as detailing the specific risk of ischemic stroke associated with estrogen use in patients with focal neurological symptoms.

### 3.2. Recommendation of Alternative Methods

When primary methods were contraindicated, all models successfully recommended appropriate alternatives, generally aligning with USMEC Category 1 or 2. For a smoker with a history of DVT (Case 1), all models recommended progestin-only pills, contraceptive implants, or IUDs. Gemini was particularly descriptive in this area, identifying the Cu-IUD as the most logical choice for patients wary of any hormonal influence. Similarly, for patients with systemic lupus erythematosus (Case 2), all models emphasized the importance of testing for antiphospholipid antibodies before initiating hormonal contraception, with OpenEvidence and Gemini correctly identifying that progestin-only methods might be rated as USMEC 3 in the presence of these antibodies.

### 3.3. Drug Interactions and Clinical Implications

The models displayed high proficiency in managing complex drug interactions, specifically the interaction between lamotrigine and estrogen (Case 5). Every model correctly noted that estrogen induces the metabolism of lamotrigine, which can reduce its serum levels and increase the risk of breakthrough seizures. OpenEvidence suggested close collaboration with neurology for dose adjustments, while Gemini and Chat-GPT recommended non-estrogen methods as the gold standard to avoid this interaction altogether. Gemini also noted that while combined hormonal contraceptives are not strictly forbidden (USMEC 3), progestin-only or nonhormonal methods are preferred for better reliability.

### 3.4. Management of Side Effects and Complications

Regarding the management of common complications, such as spotting after Cu-IUD insertion (Case 7) or amenorrhea with a contraceptive implant (Case 8), the models provided evidence-based strategies. For Case 7, all models correctly identified that spotting is common and typically not pathological in the first few weeks, recommending Nonsteroidal Anti-Inflammatory Drugs (NSAIDs) for symptomatic management. For Case 8, all models reassured the patient that amenorrhea is a common and medically safe side effect of the etonogestrel implant, occurring in approximately 20–22% of users. Gemini and Chat-GPT went further by providing “red flag” symptoms for the patient in Case 7 to monitor, such as severe pelvic pain or fever, which were less emphasized in the gold standard summary.

### 3.5. Backup Contraception and Emergency Protocols

The models provided accurate and detailed guidance on backup contraception and emergency protocols. In the case of two missed active pills (Case 9), every model correctly advised taking the most recent missed pill immediately and using backup barrier methods for seven consecutive days. For emergency contraception management following vomiting (Case 10), all models identified the 3 h absorption window and recommended an immediate repeat dose. Gemini provided the most granular practical advice, suggesting the use of an anti-emetic 30 to 60 min before the repeat dose and advising the patient to take the pill with food.

### 3.6. Patient Education and Communication Style

While technical accuracy was consistent, the models diverged in their communication approach. Chat-GPT consistently adopted a structured consultation style, including sections on “shared decision-making,” “documentation,” and the use of “simple, non-judgmental language”. It was the only model to explicitly recommend starting consultations by clarifying the patient’s goals and taking a focused medical history. OpenEvidence remained focused on delivering evidence-based summaries of MEC levels. Gemini bridged these two approaches by providing highly practical, action-oriented instructions, such as advising against the use of tampons or swimming for 24–48 h after IUD insertion to reduce infection risk (Case 6), a level of practical detail not directly found in the other models or the gold standard.

### 3.7. Overall Assessment

Across the ten predefined clinical case scenarios, all three LLMs correctly identified all mandatory safety-related counseling elements according to the predefined evaluation rubric (Table 2), corresponding to an observed accuracy of 100% (10/10 cases; 95% CI approximately 72–100%). None of the models provided clinically unsafe recommendations or failed to recognize a major contraindication. Differences between models were primarily confined to optional counseling elements. Chat-GPT most consistently incorporated shared decision-making, documentation, and patient-centered counseling, whereas Gemini more frequently included practical implementation advice. OpenEvidence generally focused on concise evidence-based recommendations while including fewer optional counseling elements. The structured assessment of each model against the predefined criteria is summarized in Table 3. Table 4 summarizes the responses of the three LLMs in each case.

## 4. Discussion

This study demonstrates that LLMs achieved uniformly high accuracy in identifying contraindications and providing evidence-based contraceptive counseling in this limited set of standardized fictitious cases. All three LLMs correctly identified primary contraindications according to MEC guidelines and provided appropriate alternative contraceptive recommendations when combined hormonal contraceptives were contraindicated. However, the study revealed important differences in communication style and practical guidance among the models, despite their equivalent technical accuracy. Chat-GPT consistently adopted a structured consultation approach emphasizing shared decision-making, documentation, and patient-centered language, while Gemini provided highly practical, action-oriented instructions. OpenEvidence maintained a more concise, evidence-focused approach that closely mirrored technical guideline language.

These findings align with recent literature demonstrating that LLMs can provide largely accurate and complete responses to patient questions in obstetrics and gynecology. A 2025 study evaluating Chat-GPT and Google Gemini across common obstetrical and gynecologic topics found that most responses met acceptable criteria for accuracy and completeness, with Chat-GPT demonstrating stronger overall outcomes [8]. Similarly, research evaluating Chat-GPT as a fertility counseling tool found that it provided responses comparable to established sources like the CDC in terms of factual content and sentiment [19]. However, the current study’s finding of high accuracy in contraindication identification represents notably stronger performance than reported in some other clinical domains. For example, a study evaluating GPT-4 and GPT-3.5 for emergency department clinical recommendations found that both models performed poorly compared to resident physicians, with accuracy scores 8% and 24% lower, respectively, and demonstrated a tendency toward overly cautious recommendations [20]. This discrepancy may reflect the structured nature of contraceptive counseling guidelines, which provide explicit, evidence-based categorization of contraceptive safety across medical conditions, creating a well-defined knowledge structure that LLMs can effectively encode and retrieve [1,6].

The primary strength of this study is its systematic evaluation of three publicly available LLMs using standardized clinical case scenarios that represent common and clinically significant situations encountered in hormonal contraception consultation, with independent assessment by two board-certified obstetrician–gynecologists using evidence-based guidelines. The case scenarios were specifically designed to assess LLM competency across multiple domains including medical eligibility screening, contraindication recognition, drug interaction counseling, side effect management, and emergency contraception guidance, providing comprehensive evaluation of LLM performance in contraceptive care. However, several important limitations must be acknowledged. First, the selected vignettes were relatively ‘guideline-friendly’ and structured around distinct, isolated medical considerations. In routine clinical practice, contraceptive consultations are rarely singular; they frequently involve complex, overlapping patient factors such as concurrent obesity, hypertension, undiagnosed thrombophilia, psychiatric medications, poor adherence histories, and shifting patient preferences. By evaluating isolated clinical conditions, our study design may over-estimate the baseline proficiency and reasoning capabilities of current LLMs. Second, the study did not explicitly assess readability and health literacy appropriateness of LLM-generated responses, which is critical for patient education. Third, the study was conducted at a single time point in January 2026 utilizing the free, publicly available versions of the selected LLMs. While this approach allows us to evaluate the tools available to the broadest public demographic without financial barriers, it does not account for premium, subscription-based models. Paid subscription tiers often utilize more advanced reasoning engines and real-time web-browsing integrations, which may yield different depths of information. Notably, as LLMs tend to adapt their responses to an individual user’s response patterns and preferences, these responses might not be generalizable to what actual users may receive as a response, especially in situations where the LLMs may be biased towards providing misinformation or confirming unfounded biases by the user. Fourth, all ten clinical case scenarios were presented sequentially within a single, continuous chat session for each model, and browser cookies or account history settings were not cleared prior to data collection. Because LLMs maintain an active context window during a single conversation, the models’ responses to later cases may have been influenced or biased by the medical concepts introduced in earlier vignettes. While this sequential prompting design closely mirrors a real-world multi-topic clinical consultation, it represents a methodological limitation as the responses cannot be considered entirely independent baseline generations. Another limitation is that formal inter-rater reliability statistics were not prospectively collected.

Future research should focus on several key areas to optimize LLM performance in contraceptive counseling and ensure safe clinical integration. Development of specialty-specific models with enhanced training on reproductive health guidelines, contraceptive pharmacology, and patient-centered counseling frameworks may improve both accuracy and communication quality, while implementation of rigorous content validation processes involving board-certified obstetrician–gynecologists and family planning specialists can help identify and correct errors before clinical deployment. Research should explore optimal integration strategies for incorporating LLMs into clinical contraceptive care workflows while maintaining appropriate clinical oversight, including evaluation of how LLMs can best support rather than replace comprehensive contraceptive counseling by healthcare professionals, as well as contribute to cost reduction in health care. Given these promising first results of the present feasibility study, future studies should include further local LLMs as well as more complex clinical cases representing the complexity of real life. While this study focused on widely used LLMs such as Chat-GPT, Google Gemini, and OpenEvidence, the rapid diversification of the AI landscape presents clear avenues for expansion. Future comparative trials should explicitly integrate emerging open-weights reasoning networks, such as DeepSeek, alongside specialized multi-modal models like the Anthropic Claude family. Benchmarking these models against identical clinical vignettes will be crucial to determining whether advanced self-correction capabilities or regional training variations yield a measurable advantage in clinical accuracy, patient safety, and communication quality during reproductive health consultations. Future research should also include comparative analyses between free and subscription-based LLMs to determine if premium models offer a measurable advantage in clinical safety and communication nuance for reproductive healthcare. Additionally, although the present study evaluated clinical accuracy and guideline concordance, health literacy represents a critical component of contraceptive counseling. Future studies should assess the readability, understandability, and actionability of LLM-generated responses, as well as patient perceptions, trust, and the impact of these tools on informed decision-making in real-world clinical settings.

## 5. Conclusions

This exploratory feasibility study suggests that current LLMs can generate guideline-concordant responses for selected hormonal contraception counseling scenarios and may serve as promising complementary educational tools to support patient information and clinician workflows. However, these findings are based on a limited number of predefined clinical cases and should not be interpreted as evidence that LLMs are sufficiently safe or reliable for independent patient counseling in routine clinical practice. LLMs cannot replace individualized clinical assessment, professional judgment, or shared decision-making between patients and healthcare professionals. Their implementation in reproductive healthcare should therefore occur under appropriate clinical oversight and be accompanied by continued validation, optimization of health literacy, robust privacy and data protection measures, and ongoing monitoring as models and clinical guidelines evolve. Larger studies including more diverse and complex clinical scenarios and real-world patient interactions are warranted to confirm the generalizability and safety of these findings.

## Figures and Tables

**Table 1:** Case vignettes.

Cases	Description
1	A 35-year-old patient wishes to be on hormonal contraception. The patient is an active smoker and has a history of deep vein thrombosis. How would you consult this patient?
2	A 29-year-old patient with systemic lupus erythematosus wishes to be on hormonal contraception. How would you consult this patient?
3	A 23-year-old breastfeeding patient wishes to start with a hormonal contraception two weeks after having given birth to her first child. How would you consult this patient?
4	A 22-year-old patient that suffers from headaches with eventual speech disturbances and motor weakness wishes to be on hormonal contraception. How would you consult this patient?
5	A 33-year-old patient suffers from epilepsy and is currently under lamotrigine therapy. She wishes to be on hormonal contraception. How would you consult this patient?
6	A 33-year-old patient intends to have an LNG-IUD inserted for hormonal contraception and asks you about the need for back-up contraception after its insertion. How would you consult this patient?
7	A 26-year-old woman presents in the emergency department with ongoing spotting since the insertion of a Cu-IUD three weeks ago. How would you consult this patient?
8	A 22-year-old patient has had Implanon inserted in August 2025 and still remains amenorrhoeic. How would you consult this patient?
9	A 34-year-old patient has missed 2 active pills of 30 µg of ethinyl estradiol in a row. How would you consult this patient?
10	A 22-year-old patient has received emergency contraception but had to vomit 1 h after taking UPA-ECP. How would you consult this patient?

**Table 2:** Evaluation rubric used for each clinical case.

Cases	Mandatory Elements	Optional Elements	Clinically Relevant Deviation
1	Contraindication for combined hormonal contraceptives (MEC category assignment) Recommendation of appropriate alternatives	Smoking cessation	Recommendation of combined hormonal contraceptives
2	Assessment of antiphospholipid antibodies Contraindication for combined hormonal contraceptives (MEC category assignment depending on disease status) Recommendation of appropriate alternatives	Recognition of thrombocytopenia as a significant risk cofactor	Failure to stratify antiphospholipid syndrome status Unconditional recommendation of combined hormonal contraceptives
3	Contraindication for combined hormonal contraceptives (MEC category assignment) Recommendation of appropriate alternatives	Breastfeeding-focused counseling	Recommendation of combined hormonal contraceptives
4	Recognition of migraine with aura Contraindication for combined hormonal contraceptives (MEC category assignment) Recommendation of appropriate alternatives	Neurology referral	Recommendation of combined hormonal contraceptives
5	Recognition of estrogen–lamotrigine interaction (including dose adaptation requirements) Recommendation of appropriate contraceptive alternatives	Neurology referral	Failure to identify estrogen–lamotrigine interaction
6	Correct backup contraception advice according to timing	Practical insertion advice	Incorrect backup recommendation
7	Exclusion of relevant differential underlying conditions	Proposal of NSAIDS	Missing red flags or inappropriate alarm
8	Exclusion of relevant differential underlying conditions	Recommendation of supportive measurements or appropriate alternatives	Unnecessary treatment or incorrect diagnosis
9	Correct missed-pill protocol Backup contraception Proposal of emergency contraception	Explore adherence	Incorrect protocol
10	Repeat dose Mention 3 h rule Delay hormonal contraception	Antiemetic advice	Failure to recommend repeat dose

**Table 3:** Structured assessment of the LLMs’ responses against the predefined criteria.

Cases	OpenEvidence	Chat-GPT	Gemini
1	Accurate	Complete	Complete
2	Complete	Accurate	Complete
3	Complete	Complete	Complete
4	Accurate	Complete	Complete
5	Complete	Complete	Complete
6	Accurate	Accurate	Complete
7	Complete	Complete	Complete
8	Complete	Complete	Accurate
9	Accurate	Accurate	Complete
10	Complete	Complete	Complete

**Table 4:** LLMs’ responses to the ten fictitious patient cases.

Cases	Gold Standard	OpenEvidence	Chat-GPT	Gemini
1	Combined hormonal contraceptive use in women with these conditions is classified as USMEC 3 or 4. Overall, contraindications should be considered to apply to the transdermal patch and the vaginal ring. Combined hormonal contraceptives with older formulations of progestins and newer progestins are associated with a comparable risk and can be recommended as equivalent options. Progestin-only pills, the contraceptive implant, or an LNG-IUD are appropriate options (USMEC 2). The USMEC allows the use of Depot-Medroxyprogesteronacetat (USMEC 2).	Combined hormonal contraceptives are contraindicated (USMEC 3 or 4). Nonhormonal or progestin-only contraceptive methods may be used. Depot-Medroxyprogesteronacetat may carry a slightly increased thrombotic risk, and its use should be considered with caution.	Start with a structured consultation by clarifying goals and context and taking relevant medical history. Explain the key safety issue clearly by using simple, non-judgmental language. Concerning combined hormonal contraceptives, the risk is unacceptable in a patient with previous DVT (WHO MEC 4). Safe contraceptive options include Cu- IUD and progestin-only methods. Depot-Medroxyprogesteronacetat can be used, but the possible small thrombotic risk should be discussed. Additionally, smoking cessation advice, shared decision-making, as well as safety net and documentation.	This patient falls into WHO MEC 4 for any contraceptive containing estrogen. Nonhormonal or progestin-only contraceptive methods may be used. Cu-IUD has no effect on blood coagulation and is the most logical choice for someone wary of any hormonal influence. Hormonal IUDs are generally considered safe for those with a history of DVT. Progestin-only pills do not carry the same clotting risks as combined ones. Depot-Medroxyprogesteronacetat is an option, though sometimes cautioned against in patients with multiple cardiovascular risk factors (like heavy smoking and age) due to its potential effect on lipid profiles. The consultation should include a holistic conversation about smoking cessation, thrombosis workup, and a discussion on how often patients want to think about their birth control.
2	USMEC contraceptive recommendations are stratified according to the presence or absence of antiphospholipid antibodies, severe thrombocytopenia, and concomitant immunosuppressive medications. Combined hormonal contraceptives are contraindicated in women with SLE and positive antiphospholipid antibodies (USMEC 4). In other SLE patients, use of combined hormonal contraceptive methods is rated USMEC 2 in the absence of other cardiovascular disease risk factors. Although progestin-only methods, including LNG-IUDs, are not considered to increase the risk of thrombosis, they are all rated USMEC 3 for SLE patients with antiphospholipid antibodies. In SLE patients without antiphospholipid antibodies, progestin-only methods are rated USMEC 2. For women with SLE complicated by severe thrombocytopenia, caution exists only for the initiation of Depot-Medroxyprogesteronacetat (USMEC 3). Many women with SLE take immunosuppressant medications, and LNG-IUDs are demonstrated to be a safe option (USMEC 2).	Combined hormonal contraceptives are contraindicated if antiphospholipid antibodies are present. Testing for antiphospholipid antibodies before initiating hormonal contraception in all women with SLE. If antiphospholipid antibodies are absent and there are no other major cardiovascular risk factors, combined hormonal contraceptives may be considered (USMEC 2). Progestin-only methods are generally considered safe for SLE patients without antiphospholipid antibodies (USMEC 2). For SLE patients with antiphospholipid antibodies, progestin-only methods are rated USMEC 3 due to the high baseline risk of thrombosis. For SLE patients with severe thrombocytopenia, caution is advised with Depot-Medroxyprogesteronacetat due to the risk of irregular bleeding.	First, structure the consultation, clarify contraceptive needs and focus on SLE history. Explain why lupus matters for contraception. Categorize the patient (clinically) into high-risk (positive antiphospholipid antibodies (with or without thrombosis) OR history of thrombosis OR active severe SLE) and lower-risk SLE (negative antiphospholipid antibodies, stable/inactive disease, no thrombotic history). Concerning combined hormonal contraceptives can be considered only for stable SLE, negative antiphospholipid antibodies (MEC 2). Progestin-only contraception does not increase lupus activity and has minimal thrombotic risk. Depot-Medroxyprogesteronacetat can be used, but the possible small thrombotic risk should be discussed, as well as some bone density concerns (especially with steroids). Cu-IUD poses no hormonal risk but may worsen menorrhagia (important if anemic). Also, emphasize on the importance of effective contraception as pregnancy during active SLE increases maternal and fetal complications. Shared decision-making and safety net and documentation are important, as well.	Before recommending any method, check for antiphospholipid antibodies. If positive antiphospholipid antibodies, the patient has a high baseline risk for blood clots. Estrogen-containing methods are USMEC 4. Even some progestin-only methods may be treated with caution (USMEC 3) depending on the clinical picture. If negative antiphospholipid antibodies, estrogen-containing methods are generally USMEC 2, provided the lupus is stable or inactive. The Cu-IUD is the safest non-hormonal option (use with caution if the patient has severe thrombocytopenia). Hormonal IUDs are highly recommended, especially for patients with heavy periods or those on blood thinners. It is generally safe regardless of antiphospholipid antibodies status. Contraceptive implants are a safe, progestin-only option that does not increase the risk of lupus flares. Combined hormonal contraceptives can be offered only to stable, antiphospholipid antibodies-negative patients. If the patient is on long-term steroids, avoid the Depot-Medroxyprogesteronacetat. Some immunosuppressants or medications for SLE complications may interact with hormonal metabolism.
3	Regardless of breastfeeding status, combined hormonal contraceptives are contraindicated during the first 21 days after giving birth because of the risk of thrombosis (USMEC 4). Progestin-only methods may be used without concern immediately after delivery in women who are not breastfeeding (USMEC 1). Intrauterine devices are also an option either immediately after placental delivery or as an interval insertion at the postpartum visit (USMEC 1). Progestin-only contraceptives suggest they have no effect on successful initiation and continuation of breastfeeding or on infant growth and development even when started immediately after giving birth.	Progestin-only contraceptives are appropriate and may be initiated at any time during the postpartum period, including two weeks after delivery. Combined hormonal contraceptives are contraindicated in the first 21 days postpartum. Patients should be informed of the theoretical concern that immediate postpartum progestin use could affect lactogenesis.	Start with a structured assessment by clarifying contraceptive needs and taking the relevant post-partum history. Explain key post-partum considerations. For the first 6 weeks, methods containing estrogen are not safe or suitable while breastfeeding (MEC 4). Progestin-only pills may be started immediately post-partum, etonogestrel implants can be inserted immediately after birth or any time after, LNG-IUD or Cu-IUD can be inserted within 48 h post-partum, or from ≥4 weeks post-partum, while Depot-Medroxyprogesteronacetat can be given from ≥6 weeks. Additionally, breastfeeding-specific counseling, shared decision-making, and safety net and follow-up are suggested.	Combined hormonal contraceptives are USMEC 4 before 21 days postpartum. Estrogen can potentially decrease milk supply if started before breastfeeding is well-established. Progestin-only hormonal contraception represents USMEC 1 highly effective safe options. If an IUD was not placed at delivery, most clinicians recommend waiting until 4–6 weeks postpartum. Inserting an IUD at 2 weeks carries a higher risk of expulsion and a slightly higher risk of perforation. Since the patient is breastfeeding, she may already have some natural protection, but only if she meets all three criteria of exclusive breastfeeding, amenorrhea, and the baby is less than 6 months old.
4	At the time of contraceptive initiation, the diagnosis of migraine with or without aura should be carefully considered. There are no contraindications to the use of any progestin-only method in women with migraines with or without aura (USMEC 1). Combined hormonal contraceptives can be used in women who have migraine without aura and no other risk factors for stroke (USMEC 2). Estrogen-containing contraceptives are not recommended for women who have migraine with aura because of the increased risk of stroke (USMEC 4).	Combined hormonal contraceptives are contraindicated. Symptoms are consistent with migraine with aura, and estrogen-containing contraceptives significantly increase the risk of ischemic stroke in this population (USMEC 4). Progestin-only contraceptives are recommended for patients with migraine with aura, as they do not increase stroke risk and can be used without restriction (USMEC 1).	Clarify the diagnosis (first priority). Explain why this matters for contraception as certain types of migraine increase the risk of stroke, and some hormonal contraceptives can raise that risk further. Any contraception containing estrogen is contraindicated (MEC 4). Progestin-only contraception and Cu-IUD do not increase stroke risk and are safe in migraine with aura. Additional counseling points should include neurological safety and migraine course. Shared decision-making and safety net and documentation are important, as well.	Estrogen-containing contraceptives further increase this stroke risk. Migraine with focal neurological symptoms is a USMEC 4 for any estrogen-containing method. Since estrogen is the contraindication, progestin-only and non-hormonal methods are the recommended paths. These do not increase the risk of stroke. Depot-Medroxyprogesteronacetat is safe regarding stroke risk, though some patients find that the fluctuation in hormones can occasionally affect headache frequency. A neurological consultation is also necessary.
5	Use of combined hormonal contraceptives in the setting of hepatic enzyme-inducing antiepileptic drugs is classified as USMEC 3. Use of progestin-only pills with antiepileptic drugs that induce hepatic enzymes is rated USMEC 3. Depot-Medroxyprogesteronacetat and IUDs represent two preferred contraceptives for women taking liver enzyme-inducing antiepileptic drugs (USMEC 1). The etonogestrel implant is rated USMEC 2 for women on enzyme-inducing antiepileptic medications. Lamotrigine is the only antiepileptic medication known to have its metabolism affected by estrogen-containing contraceptives, which reduces lamotrigine serum levels with concomitant use. Therefore, dose adjustments of lamotrigine may be needed.	Combined hormonal contraceptives can significantly reduce lamotrigine serum concentrations, increasing the risk of breakthrough seizures. If combined hormonal contraceptives are used, close collaboration with neurology is required for lamotrigine dose adjustment, and extended-cycle or continuous combined hormonal contraceptive regimens may be considered to avoid fluctuations. Progestin-only methods and nonhormonal options are preferred for patients on lamotrigine, as they do not interact with lamotrigine metabolism and maintain both contraceptive and antiepileptic efficacy.	Structured assessment including clarification of contraceptive needs and focus on epilepsy-specific history. Explain the key interaction clearly as estrogen induces lamotrigine glucuronidation and reduces lamotrigine levels by up to 50–60%. Lamotrigine does not reduce contraceptive efficacy. We usually avoid estrogen-containing contraception with lamotrigine unless there is a strong reason and close monitoring. LNG-IUD or Cu-IUD, etonogestrel implant and Depot-Medroxyprogesteronacetat are the preferred contraceptive options. Progestin-only pills might slightly reduce lamotrigine levels possible which is usually not clinically relevant. Additional counseling points should be seizure safety and pregnancy planning. Shared decision-making and safety net and documentation are important, as well.	Estrogen induces the liver enzymes that metabolize lamotrigine. To avoid this interaction, methods that do not contain estrogen are the gold standard for this patient. Cu-IUD is hormone-free and has zero interaction with lamotrigine. Hormonal IUD have local progestin levels do not significantly affect lamotrigine metabolism. Contraceptive implants or Depot-Medroxyprogesteronacetat are generally considered safe and highly effective for patients on lamotrigine. Progestin-only pills do not interact with lamotrigine, but they have a narrow margin for error. Since some other anti-epileptic drugs (though not lamotrigine specifically) can lower the pill’s efficacy, many neurologists prefer IUDs/implants for better reliability. If the patient strongly prefers the combined oral contraceptive pill, it is not strictly “forbidden” (USMEC 3).
6	The LNG-IUD may be placed at any time if it is reasonably certain that the patient is not pregnant. If the LNG-IUD is placed within the first 7 days since menstrual bleeding started, no additional contraceptive protection is needed. If the LNG-IUD is placed >7 days since menstrual bleeding started, the patient needs to abstain from sexual intercourse or use barrier methods for the next 7 days.	A back-up contraceptive method is recommended for 7 days after LNG-IUD insertion unless the device is inserted within the first 7 days of menstrual bleeding, immediately after surgical abortion, within 21 days postpartum, or when transitioning directly from another reliable contraceptive method.	Start by clarifying timing and pregnancy risk. If the IUD is inserted in the first week of the period, no back-up is needed. Otherwise a 7-day back-up is necessary. If inserted over 4 weeks post-partum and not menstruating, use back-up for 7 days. If switching from combined pill/progestin-only pill/implant/injection, no back-up if inserted correctly while still covered. Otherwise, use back-up for 7 days. If unprotected intercourse occurred within the previous 7 days and insertion is planned after day 7, exclude pregnancy, advise back-up contraception and discuss emergency contraception if appropriate. Shared decision-making and safety net and documentation are important, as well.	If the IUD is inserted within the first 7 days of her menstrual cycle, she is protected immediately. No back-up method is required. If the IUD is inserted after day 7, it is not considered immediately effective. She must use a back-up method or abstain from intercourse for the next 7 days. If the patient is transitioning from oral contraceptives, she should continue taking her pills for 7 days after the IUD is inserted. If the IUD is inserted within 13 weeks of a last shot of the Depot- Medroxyprogesteronacetat, she is protected immediately. If a new IUD is inserted the same day the old one is removed, she remains protected throughout. Beyond back-up contraception, many clinicians recommend avoiding tampons, swimming, or intercourse for the first 24 to 48 h simply to reduce the risk of pelvic infection while the cervix is slightly open. Moreover, the patient should check the strings once a month after her period and be aware of expected side effects such as spotting and cramping.
7	Before Cu-IUD placement, provide counseling about potential changes in bleeding patterns during Cu-IUD use. Spotting or light bleeding and heavy or prolonged bleeding is common during the first 3–6 months of Cu-IUD use, is generally not harmful but might be bothersome to the patient, and generally decreases with continued Cu-IUD use. If clinically indicated, consider an underlying health condition, such as Cu-IUD displacement, STIs, pregnancy, thyroid disorders, or new pathologic uterine conditions (e.g., polyps or fibroids), especially in patients who have already been using the Cu-IUD for a few months or longer and who have developed a new onset of heavy or prolonged bleeding. Explore patient goals, including continued Cu-IUD use (with or without treatment for bleeding irregularities) or Cu-IUD removal. If the patient wants to continue Cu-IUD use, provide reassurance, discuss options for management of bleeding irregularities if it is desired, and advise the patient that they may contact their provider at any time to discuss bleeding irregularities or other side effects. If the patient desires Cu-IUD removal at any time, remove the Cu-IUD, offer counseling on alternative contraceptive methods, and initiate another method if it is desired. If the patient wants treatment, NSAIDs may be offered as short-term treatment.	Ongoing spotting three weeks after Cu-IUD insertion is a common side effect and does not typically indicate pathology. The society further states that NSAIDs are effective for managing bothersome bleeding or cramping associated with Cu-IUDs. If the spotting is not accompanied by severe pain, fever, or signs of infection, reassurance and symptomatic management with NSAIDs are appropriate.	Start with a focused assessment to rule out complications and actively exclude red flags (severe or increasing pelvic pain, fever, heavy bleeding, positive pregnancy test). Examination and investigations as appropriate in ED. Spotting and irregular bleeding are very common in the first weeks after a copper coil is inserted as Cu-IUD causes a local inflammatory reaction. Conservative management includes reassurance and expectant management. Symptomatic treatment includes NSAIDs and tranexamic acid if bleeding is bothersome (short course). Shared decision-making and safety net and documentation are important, as well.	At first, pregnancy, infection and displacement/perforation should be ruled out. If the pregnancy test is negative, the strings are in the right place, and there are no signs of infection, the spotting is likely a normal physiological response to the Cu-IUD. The Cu-IUD works by causing a local inflammatory response in the uterus. This often leads to increased spotting, heavier periods, and more intense cramping during the first 3 to 6 months. If the spotting is bothersome to the patient, we can offer NSAIDs. Before leaving the ED, I would provide clear “Red Flag” symptoms that require her to return immediately.
8	Before implant placement, provide counseling about potential changes in bleeding patterns during implant use. Bleeding changes are generally not harmful but might be bothersome to the patient. Bleeding changes might or might not decrease with continued implant use. Amenorrhea does not require any medical treatment. If a patient’s regular bleeding pattern changes abruptly to amenorrhea, consider ruling out pregnancy if clinically indicated. If the patient desires implant removal, remove the implant, offer counseling on alternative contraceptive methods, and initiate another method if it is desired.	Amenorrhea is a common and expected side effect of the etonogestrel implant, occurring in approximately 20–22% of users, and does not indicate any health risk or loss of contraceptive efficacy. Menstrual cycle changes, including amenorrhea, are normal with progestin-only implants, and no treatment is required unless the symptom is bothersome. If the patient desires regular bleeding or is concerned, options for managing bothersome bleeding include add-back therapy with continuous combined oral contraceptives or norethindrone acetate, but this is not necessary for amenorrhea alone. If there are risk factors or symptoms suggestive of pregnancy or endocrine disorders, consider ruling out other causes of amenorrhea.	Start by understanding the concern. Exclude pregnancy. Explain why amenorrhoea happens with etonogestrel implant. Assess whether amenorrhoea is acceptable. Short course of combined oral contraceptive (if no contraindications) or NSAIDs if bleeding is desired. Shared decision-making and safety net and documentation are important, as well.	Based on clinical data, amenorrhea is a very common, expected, and medically safe side effect of this contraceptive method. It is important to explain why her period has stopped so she understands it is not a sign of a problem. The patient is actually in a significant group of implant users. Even though amenorrhea is expected, a quick “safety check” is still appropriate, including ruling out pregnancy and checking for other symptoms. For many patients, amenorrhea is actually considered a “non-contraceptive benefit”, including convenience, pain reduction, and anemia prevention.
9	Take the most recent missed pill as soon as possible. Continue taking the remaining pills at the usual time. Abstain from sexual intercourse or use barrier methods for 7 consecutive days. If pills were missed during the last week of hormonal pills, omit the hormone-free interval. Consider emergency contraception if hormonal pills were missed during the first week and unprotected sexual intercourse occurred during the previous 5 days.	Missing two active pills reduces contraceptive efficacy and increases the risk of unintended pregnancy, especially if unprotected intercourse occurs in the 7 days following the missed pills. If the missed pills occurred in Week 1 or Week 2 of the pack, the patient should take two pills on the day remembered and two pills the next day, then resume one pill daily until the pack is finished. If the missed pills occurred in Week 3, the management depends on the pill start regimen: for Day 1 starters, discard the rest of the pack and start a new pack that same day; for Sunday starters, continue taking one pill daily until Sunday, then discard the rest of the pack and start a new pack on Sunday. Regardless of the week, back-up contraception is required for 7 days after the missed pills. If unprotected intercourse occurred in the 7 days after missing pills, emergency contraception may be considered.	Start by clarifying key details. If the patient misses two or more active pills, she should take action straight away and use back-up contraception. Regardless of week, take the most recent missed pill immediately, discard earlier missed pill(s), continue the rest of the pack as normal and use condoms or abstain for the next 7 days. Emergency contraception is indicated in case the patient missed 2 pills in week 1 and had unprotected sex in last 7 days. In week 2, no emergency contraception needed if pills before were taken correctly. In week 3, risk is loss of hormone cover before the pill-free interval. Contraceptive protection resumes after 7 consecutive active pills. Shared decision-making and safety net and documentation are important, as well.	Take the most recent missed pill now. Even if it means taking two pills in one day (the one she just remembered and today’s scheduled pill). Discard the other missed pill. She should finish the rest of the pack on her usual schedule, one pill per day. She must use a back-up method or abstain from sex until she has taken the active hormonal pills for 7 consecutive days. If the misses occurred in Week 1 and had unprotected intercourse in the last 5 days, she should strongly consider taking Emergency Contraception. If the misses occurred in Week 2, as long as she took the pills correctly for the 7 days before the missed pills, the risk is lower, but the 7-day back-up rule still applies for future protection. If the misses occurred in Week 3, to prevent a drop in hormones before the next pack, she should skip the placebo pills. She should finish the active pills in the current pack and start the active pills of a new pack immediately the next day. I would ask the patient why the pills were missed. If she finds it difficult to remember a daily pill, this is a great time to discuss LARC like an IUD or the Implant, which removes the risk of human error.
10	Another dose of ECP should be taken as soon as possible. Use of an antiemetic should be considered. Start or resume hormonal contraception no sooner than 5 days after use of UPA.	If vomiting occurs within 3 h of taking UPA-ECP, the dose may not have been absorbed and a repeat dose should be considered; the patient should contact their healthcare provider promptly to discuss re-dosing. Advise the patient to use a reliable barrier method of contraception until the next menstrual period, and to delay starting or resuming hormonal contraception for at least 5 days after UPA intake. Counsel that menstrual timing may be affected, with menses possibly occurring earlier or later than expected; if the period is delayed by more than 1 week, pregnancy should be ruled out. Inform the patient that nausea and vomiting are common side effects of emergency contraception, and that UPA does not protect against sexually transmitted infections. Offer antiemetics for patients at high risk of vomiting.	Acknowledge and reassure. Vomiting within 3 h of taking UPA-ECP means that the dose is not reliable. At 1 h, absorption is very unlikely to be sufficient. Give another dose of UPA-ECP as soon as possible. Consider giving an antiemetic first. This is appropriate if still within 120 h (5 days) of unprotected intercourse. Cu-IUD is the most effective emergency contraception and can be inserted up to 5 days after ovulation, or 5 days after unprotected intercourse. Do not switch to LNG-IUD EC if UPA has already been used. Ongoing contraception advice. Shared decision-making and safety net and documentation are important, as well.	The absorption window for emergency contraceptive pills is generally 3 h. The patient needs to repeat the dose as soon as possible to ensure the window for preventing ovulation has not closed. She should take another 30 mg UPA tablet immediately. To prevent a second episode of vomiting, I would recommend taking an anti-emetic like meclizine or metoclopramide 30 to 60 min before the repeat dose and taking the pill with a small amount of food. If the patient is concerned about nausea or if this is her second attempt, we should discuss the most effective form of emergency contraception such as the Cu-IUD. In addition, she should avoid progestin for 5 days and use condoms for at least 7 days.

## Data Availability

The authors confirm that the key data supporting the findings of this study are available within the article and its accompanying tables. Due to the extensive length of the raw AI generations, the complete, unedited prompts and full verbatim responses from each evaluated LLM are available from the corresponding author upon reasonable request.
